# The Blood in Hodgkin's Disease

**Published:** 1907-04-27

**Authors:** 


### Points in Diagnosis.

### THE BLOOD IN HODGKIN'S DISEASE.

It frequently happens that specimens of blood are sent to the laboratory for investigation with some such query as the following: "My patient has some very enlarged glands in the neck, and in one axilla; I think the trouble is either tuberculous or else due to Hodgkin's disease; will you please examine the blood and tell me if it indicates Hodgkin's disease?"

### Positive and Negative Points.

This query is chiefly made by the surgeon, who wishes to decide whether the glands should be excised or not. From the frequency with which it is made, it would seem that there is a widespread belief that the blood in Hodgkin's disease presents features of a *positive* nature. This, of course, is not the case; its features are all *negative*. A blood examination in Hodgkin's disease is quite necessary, in order to exclude lymphatic leuchæmia and spleno-medullary leuchæmia, in both of which the blood changes are positive (see article on "Leuchæmia," already published). It would also help to exclude simple inflammation of the glands with tendency to abscess formation, for in such a case there would be leucocytosis with relative increase in the polymorphonuclear cells (see article on "Differential Leucocyte Counts"). Beyond excluding these possibilities, however, it is important to remember that the blood examination in Hodgkin's disease assists but little in the diagnosis, and certainly does not serve to distinguish this disease from tuberculous glands.

### What the Blood-count Shows.

In the early stages of the disease the blood is that of an apparently normal person; the red corpuscles at this time may still number 5,000,000 per cubic millimetre, the hæmoglobin may be from 80 per cent. to 100 per cent. of normal; the leucocytes may number 6,000 per cubic millimetre; the differential leucocyte-count may show no departure from that of health; and in stained films the red blood discs may be of fairly uniform size and shape, staining well, and presenting no nucleated varieties.

As the patient becomes more ill, anæmia of the chlorotic type will supervene. The hæmoglobin will diminish before the red corpuscles do so, the colour-index (*i.e.*, the ratio of hæmoglobin to red corpuscles) falling below 1.0. Even when the illness is advanced, there is seldom leucocytosis, and the differential leucocyte-count shows no constant type. It is not uncommon to find a moderate increase in the small lymphocytes relatively to the other white cells, the polymorphonuclear cells being proportionately diminished; on the other hand, there are cases in which the reverse occurs, the polymorphonuclear cells being relatively increased and the small lymphocytes diminished.

### The Presence of Myelocytes.

If there is any help to be expected from the differential leucocyte-count at all, it is that it will very likely show the presence of a small number of myelocytes, 1 or 2 per cent., for example, and an occasional basophile cell. The myelocytes and basophile cells by no means prove that the condition is Hodgkin's disease, but they indicate that there is something distinctly wrong with the blood, and therefore tend to support a diagnosis of Hodgkin's disease made upon other grounds.

### Two Typical Counts.

The following specimens of differential leucocyte-counts, both from typical cases of Hodgkin's disease, show how inconstant the figures may be, and therefore how little they serve as a basis for differential diagnosis:—

*Hodgkin's Disease with Relative Increase in the Small Lymphocytes.*

(*Total leucocytes* 7,200 *per cubic millimetre of blood.*)

| | per cent. |
|---|---|
| Small lymphocytes ... ... ... ... ... | 42 |
| Large hyaline lymphocytes ... ... | 6 |
| Polymorphonuclear cells ... ... ... | 50 |
| Coarsely granular eosinophile cells ... | 1.5 |
| Myelocytes ... ... ... ... ... ... | 0.5 |
| Basophile cells ... ... | an occasional one seen |

*Hodgkin's Disease with Relative Increase in the Polymorphonuclear Cells.*

(*Total leucocytes* 5,600 *per cubic millimetre of blood.*)

| | per cent. |
|---|---|
| Small lymphocytes ... ... ... ... | 14 |
| Large hyaline lymphocytes ... ... | 5.5 |
| Polymorphonuclear cells ... ... | 77 |
| Coarsely granular eosinophile cells | 1.7 |
| Myelocytes ... ... ... ... ... | 1.2 |
| Basophile cells ... ... ... ... ... | 0.6 |

### Points in Favour of Hodgkin's Disease.

When there is an enlarged spleen as well as enlarged lymphatic glands, an absence of leucocytosis is a strong argument in favour of Hodgkin's disease, whatever the rest of the blood-count may show; the difficulty in diagnosis chiefly arises when there are enlarged glands, but no enlarged spleen. It is the purpose of the present article to emphasise the fact that the blood examination in such a case will not permit one to say definitely "This *is* a case of Hodgkin's disease"; the most that can be expected from the blood-count is the exclusion of a few of the other possibilities; so that, when the blood shows no particular characteristics, one may say "The condition of this blood *is compatible with* a diagnosis of Hodgkin's disease."

